# Monitoring Antipsychotic Adherence in a Community Depot Clinic

**DOI:** 10.1192/bjo.2024.496

**Published:** 2024-08-01

**Authors:** Ayoub Behbahani, Catherine Willis, Kuben Naidoo

**Affiliations:** Mersey Care, Liverpool, United Kingdom

## Abstract

**Aims:**

In conditions such as schizophrenia insight may be limited, leading to partial adherence to antipsychotic medication. This can result in lower remission rates in this group and increased disease burden. Depot injections allow close monitoring of treatment adherence and early intervention where needed.

We aimed to determine the treatment adherence of patients attending the outpatient depot clinic at the South Sefton Neighbourhood Centre (SSNC) for antipsychotic injections and compare adherence between depot medication administered at 1, 2, 3 and 4 weekly intervals.

**Methods:**

We identified patients attending the depot clinic at the SSNC using depot cards. The RIO patient electronic record was used to find previous depot cards and to record the number of doses given each month and calculate the number of failed encounters over a twelve-month period.

We excluded patients receiving the injection at home and those where 12 months of data could not be collected.

**Results:**

42 (12 female, 30 male) patients were included. 18 had full adherence and 24 had partial adherence. Average adherence was 93%; 90% in the female group and 94% in the male group. We compared adherence to weekly (7 patients), 2 weekly (15 patients), 3 weekly (8 patients) and 4 weekly (12 patients) depot injections. Weekly and 2 weekly had an average adherence of 89%, while 3 and 4 weekly had an average adherence of 96% and 99% respectively. The average number of failed encounters was highest with the 2 weekly group and lowest in the 3 and 4 weekly group.

**Conclusion:**

Adherence to antipsychotic depot treatment at SSNC is good with nearly half of the patients included having full adherence. 4 weekly depot injections showed the best adherence with an average of 99%. Following on from this study we would like to explore the reasons for partial adherence in the two weekly group as well as the impact this has had on this group of patients, looking specifically at relapse and readmission rates.

